# ROCK inhibition promotes microtentacles that enhance reattachment of breast cancer cells

**DOI:** 10.18632/oncotarget.3360

**Published:** 2015-01-31

**Authors:** Lekhana Bhandary, Rebecca A. Whipple, Michele I. Vitolo, Monica S. Charpentier, Amanda E. Boggs, Kristi R. Chakrabarti, Keyata N. Thompson, Stuart S. Martin

**Affiliations:** ^1^ University of Maryland School of Medicine, Marlene and Stewart Greenebaum National Cancer Institute Cancer Center, University of Maryland, School of Medicine, Baltimore, Maryland, USA; ^2^ Graduate Program in Molecular Medicine, University of Maryland, School of Medicine, Baltimore, Maryland, USA; ^3^ Department of Physiology, University of Maryland, School of Medicine, Baltimore, Maryland, USA

**Keywords:** ROCK, microtentacles, Y-27632, breast cancer metastasis, reattachment

## Abstract

The presence of circulating tumor cells (CTCs) in blood predicts poor patient outcome and CTC frequency is correlated with higher risk of metastasis. Recently discovered, novel microtubule-based structures, *microtentacles*, can enhance reattachment of CTCs to the vasculature. Microtentacles are highly dynamic membrane protrusions formed in detached cells and occur when physical forces generated by the outwardly expanding microtubules overcome the contractile force of the actin cortex. Rho-associated kinase (ROCK) is a major regulator of actomyosin contractility and Rho/ROCK over-activation is implicated in tumor metastasis. ROCK inhibitors are gaining popularity as potential cancer therapeutics based on their success in reducing adherent tumor cell migration and invasion. However, the effect of ROCK inhibition on detached cells in circulation is largely unknown. In this study, we use breast tumor cells in suspension to mimic detached CTCs and show that destabilizing the actin cortex through ROCK inhibition in suspended cells promotes the formation of microtentacles and enhances reattachment of cells from suspension. Conversely, increasing actomyosin contraction by Rho over-activation reduces microtentacle frequency and reattachment. Although ROCK inhibitors may be effective in reducing adherent tumor cell behavior, our results indicate that they could inadvertently increase metastatic potential of non-adherent CTCs by increasing their reattachment efficacy.

## INTRODUCTION

Cytoskeletal rearrangements within tumor cells play important roles during cancer metastasis, which is the leading cause of patient mortality [1]. In addition to the reorganization of the cytoskeleton enabling once stationary, adherent tumor cells to migrate and invade [2], cell deformability is necessary for the survival of disseminated tumor cells that enter circulation [3]. The Rho family of small GTPases are critical mediators of tumor cell plasticity and regulate the cytoskeleton resulting in changes in cell morphology, polarity and migration [4]. Over-expression or activating mutations in Rho and its downstream effector ROCK [5] have been reported in a number of cancers, including breast [6], bladder [7] and brain cancer [8]. Moreover, the Rho/ROCK pathway has been implicated in tumor cell migration and invasion via its roles in tail retraction of migrating cells, focal adhesion turnover and invadopodia formation [7, 9].

Growth factor binding and/or integrin clustering activates Rho and ROCK resulting in increased cellular contraction and actin filament stabilization through a series of downstream phosphorylation events [4]. ROCK, a serine/threonine protein kinase, regulates contraction by activating non-muscle myosin II, through direct phosphorylation of myosin II [10] as well as by phosphorylating and inactivating the myosin dephosphorylating enzyme, myosin phosphatase [11]. Increased bundling of actin by myosin as a result of Rho/ROCK activation leads to cellular contraction and formation of actomyosin stress fibers in the cytosol that terminate at the plasma membrane in focal adhesions [11]. ROCK further stabilizes actin filaments by inactivating the actin severing protein, cofilin [12]. In addition to modulating the actin cortex, the Rho/ROCK signaling pathway has bifurcating roles in the regulation of microtubule stability [13]. While activation of Rho GTPase can increase stable detyrosinated microtubules through its downstream effector mDia [14], it can also destabilize microtubules through ROCK mediated inactivation of tubulin-stabilizing protein tau [15] and inhibition of tubulin stabilizing post-translational modification acetylation [16]. Furthermore, the microtubule cytoskeleton can in turn regulate Rho signaling since depolymerization of microtubules releases the microtubule-bound RhoGEF-H1, resulting in an increase in Rho-mediated stress fiber formation and actomyosin contractility [17, 18].

A number of recent studies using cancer cell-lines and mouse metastasis models have shown that inhibition of ROCK activity has been effective in reducing tumor progression [9, 19-21], which has generated significant interest in using ROCK inhibitors as cancer therapies. However, the effect of these drugs on detached and disseminated tumor cells that are in circulation is largely unknown. The presence of circulating tumor cells (CTCs) in blood is associated with decreased disease-free and overall survival in patients with primary as well as metastatic disease [22, 23]. Hematogenous dissemination of tumor cells can occur even before the primary tumor is detected and surgical resection of tumors can increase CTCs 1000-fold [24, 25]. Although anoikis or blood flow shear forces usually destroy epithelial cells that enter circulation, homotypic and heterotypic cell aggregations promote CTC survival and extravasation that can lead to metastatic outgrowth [3].

Given that previous studies measuring the efficacy of ROCK inhibitors as a chemotherapeutic have focused only on cells that are attached to the extra-cellular matrix, we wanted to investigate the effects of ROCK inhibition in tumor cells that are detached and free-floating in order to mimic disseminated tumor cells in circulation. We found inhibiting ROCK activity in suspended breast cancer cells increased the formation of microtubule-based structures, termed *microtentacles* (McTNs). McTNs are highly dynamic membrane protrusions formed in cells detached from the extra-cellular matrix [26] and occur when physical forces generated by the outwardly expanding microtubules overcome the inward contractile force of the actin cortex [27]. Previously published studies from our laboratory have shown that McTNs promote CTC aggregation [26], reattachment to the endothelium [28] and CTC lung retention [29, 30] in mice. This study demonstrates that ROCK inhibition results in a destabilized actin cortex that is unable to resist the formation of McTNs. The increase in McTNs corresponds with an increased reattachment efficacy of suspended breast tumor cells *in vitro*. Conversely, over-activating the Rho/ROCK pathway results in a more contractile and stabilized actin cortex resulting in a decrease in McTNs and *in vitro* reattachment. These findings indicate that inhibiting ROCK activity can give disseminated breast tumor cells a reattachment advantage by increasing the formation of McTNs.

## RESULTS

### Inhibiting ROCK activity decreases actomyosin interaction in adherent and suspended cells

**Figure 1:**
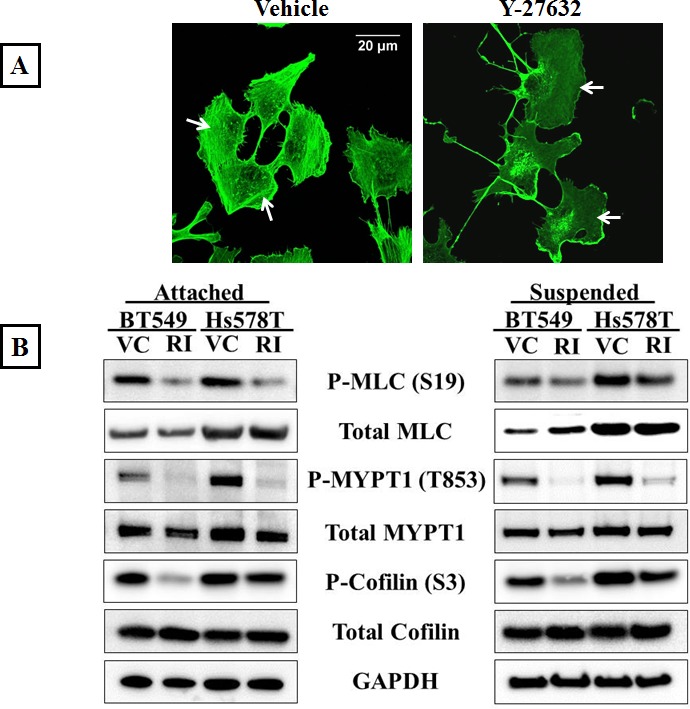

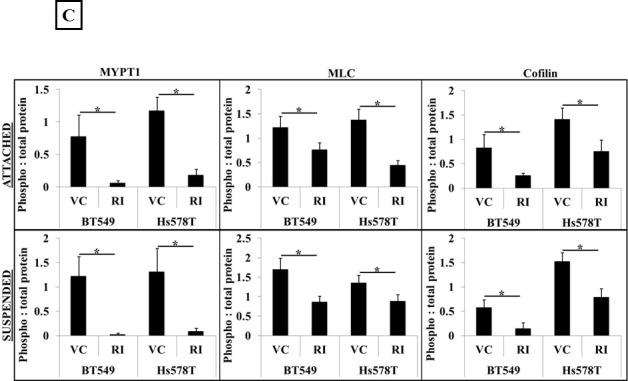
Inhibition of ROCK activity decreases actomyosin interaction A) Vehicle-treated BT549 cells stained with phalloidin, a filamentous actin binding dye, show the presence of actomyosin stress fiber bundles in the cell cytosol and periphery (arrows); 10μM Y-27632 treatment for 1 hour results in a loss of stress fiber formation in the BT549 cells (arrows). B) Representative western blot images (N=3) of attached and suspended BT549 and Hs578T cell lysates show a decrease in the phosphorylation status of myosin (MLC), the myosin-binding subunit of myosin phosphatase (MYPT1) and cofilin after 1 hour of 10μM Y-27632 treatment [RI], while the total protein levels are comparable to vehicle-treated controls [VC]. C) Densitometric analysis of 3 independent experiments shows a statistically-significant decrease in levels of phosphorylated MLC, MYPT1 and cofilin in both cell lines, BT549 and Hs578T (p<0.05, t-test, black asterisks).

The Rho/ROCK signaling pathway is required for actomyosin crosslinking and inhibition of ROCK activity decreases cellular contraction [31]. In order to assess the effects of inhibiting ROCK in metastatic breast cancer cell lines, BT549 and Hs578T, the cells were treated with the compound Y-27632, an ATP competitive inhibitor of ROCK activity [32]. Stress fibers are actin filament bundles cross-linked by myosin that require active ROCK for their formation and thus serve as a downstream indicator of ROCK activity. To visualize stress fibers, the cells were stained with phalloidin, a filamentous actin binding dye. Immunofluorescence analysis showed that vehicle-treated BT549 cells displayed thick actin stress fibers in the cell center as well as the cell periphery (Figure 1A-Vehicle). Treatment with 10μM Y-27632 for one hour resulted in a complete loss of stress fibers throughout the cell body confirming ROCK inhibition (Figure 1A-Y-27632). Since ROCK regulates the actin cytoskeleton through phosphorylation of its downstream substrates, to determine the efficacy of ROCK inhibition we analyzed protein phosphorylation levels in the Y-27632 treated cells. Myosin II is a motor protein that forms a closed compact molecule due to head to tail interactions (known as the assembly incompetent form) [33]. ROCK-mediated phosphorylation of the regulatory light chain of myosin (MLC) at serine-19 unfolds myosin into an assembly-competent conformation that is capable of binding actin. ROCK further regulates myosin phosphorylation by inactivating myosin phosphatase (MLCP) through phosphorylation of its myosin-binding subunit (MYPT1) at threonine-853, which prevents MLCP from binding to myosin [11]. Western blot analysis showed that Y-27632 treatment decreased the levels of phosphorylated MLC as well as phosphorylated MYPT1, while the total levels of both proteins were comparable to those in vehicle-treated cells (Figure 1B). This decrease in the assembly competent form of myosin indicates decreased actin binding and bundling activity. Additionally ROCK can stabilize the actin cortex by phosphorylating and inactivating cofilin [12]. Y-27632 treatment decreased phosphorylated cofilin in both cell lines while total protein levels of cofilin remained the same, indicating there is more dephosphorylated (active) cofilin that can destabilize actin filaments upon ROCK inhibition (Figure 1B).

We wanted to analyze if the results of ROCK inhibition seen in adherent cell conditions could be extended to cells that are detached. Our rationale was that disseminated cancer cells in circulation do not experience cell-cell or cell-ECM contacts and to predict the effect of ROCK inhibition in these cells, we have to mimic the free-floating microenvironment of CTCs. In order to achieve this, BT549 and Hs578T cells were suspended in low-attach plates in full-serum containing media and treated with 10μM Y-27632 for one hour. Western blot analysis showed similar decreases in levels of phosphorylated cofilin, myosin and myosin phosphatase as observed in adherent cell conditions, indicating that cells in suspension also have decreased actomyosin interaction and cortical stabilization when treated with Y-27632 (Figure 1B). Densitometric analysis of 3 independent experiments confirmed the statistical significance and consistency of these results (Figure 1C). Cell viability studies confirmed that the observed results were not confounded by drug related cytotoxicity (Supplementary Figure 1A&B).

### Over-activation of Rho signaling pathway increases actomyosin interaction in adherent and suspended cells

**Figure 2:**
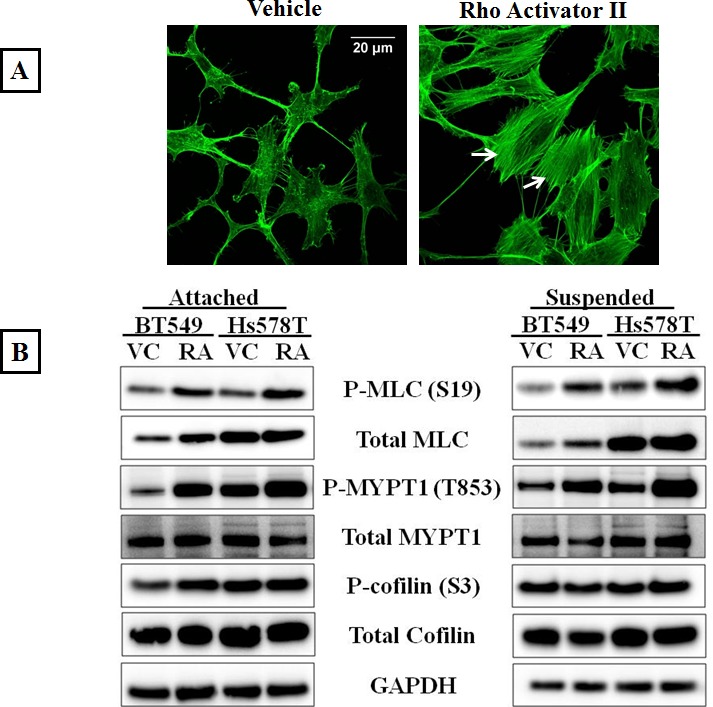

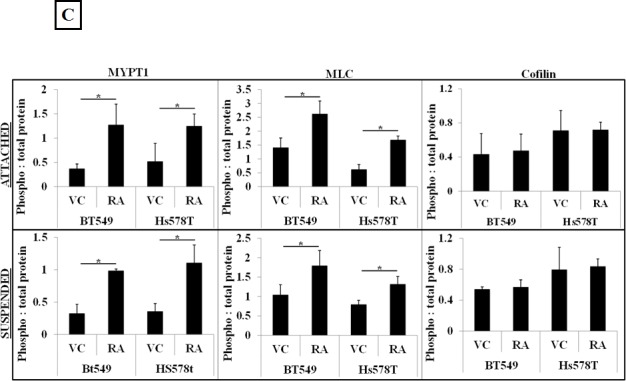
Rho over-activation increases actomyosin interaction A) Serum starved, vehicle-treated BT549 cells stained with the filamentous actin binding dye, phalloidin show lack of stress fibers due to low endogenous Rho activity; treatment of BT549 cells with the Rho activator, Rho Activator II (4μg/ml, 3 hours) results in robust stress fiber formation (arrows). B) Representative western blot images (N=3) of attached and suspended BT549 and Hs578T cell lysates show an increase in the phosphorylation status of myosin (MLC) and the myosin-binding subunit of myosin phosphatase (MYPT1) with 4μg/ml Rho Activator II treatment for 3 hours [RA], while total protein levels are comparable to vehicle-treated controls [VC]. C) Densitometric analysis of 3 independent experiments shows a statistically significant increase in levels of phosphorylated MLC and MYPT1 but no significant change in the levels of phosphorylated cofilin between vehicle and 4μg/ml Rho Activator II treated cells (p<0.05, t-test, black asterisks).

To determine if over-activating the Rho/ROCK pathway would increase actomyosin cross-linking, a Rho pathway activator (Rho Activator II) was used. Rho Activator II deamidates glutamine-63 of Rho, which prevents GTP hydrolysis and keeps Rho in its active GTP-bound state [34]. Since serum in media used to culture cells contains Rho activating components [35], BT549 and Hs578T cells were serum starved for 24 hours prior to drug treatment to obtain baseline values for Rho activity. Immunofluorescence analysis of the vehicle-treated BT549 cells stained with phalloidin showed a lack of stress fibers due to low endogenous Rho/ROCK activity as a result of serum starvation (Figure 2A-Vehicle) Serum starved BT549 cells treated with 4μg/ml Rho Activator II for 3 hours displayed thick stress fibers throughout the cell body (Figure 2A-Rho Activator II). In corroboration with the increased stress fibers observed by immunofluorescence, protein analysis of cells treated with Rho Activator II showed increased phosphorylated myosin as well as phosphorylated inactive myosin phosphatase compared to vehicle control treated cells, suggesting that in the Rho Activator II-treated cells myosin is in its assembly-competent form that can crosslink and bundle actin filaments (Figure 2B).

To determine the effect of Rho pathway over-activation on free-floating detached cells, the cells were serum starved for 24 hours and plated in ultra-low attachment plates and treated with 4μg/ml Rho Activator II for 3 hours. As observed in the adherent cell conditions, Rho Activator II treatment similarly increased phosphorylation of MLC and MYPT1 proteins in suspended cells (Figure 2B). ROCK promotes actin stabilization by phosphorylation and inactivation of cofilin, thereby preventing actin filament severing [12]. Unexpectedly, Rho Activator II treatment did not show an increase in the phosphorylation status of cofilin in adherent or suspended conditions, indicating that Rho over-activation does not result in a significant change in cofilin phosphorylation. However, under these conditions the cells already have high levels of phosphorylated (inactive) cofilin that cannot destabilize actin. Perhaps the cofilin inactivation is saturated and the cells are incapable of further phosphorylating this protein. Densitometric analysis of 3 independent experiments confirmed the statistical significance and consistency of these results (Figure 2C). The increased stress fiber formation, phosphorylation of myosin and myosin phosphatase indicates that 4ug/ml Rho Activator II effectively over-activates the Rho pathway resulting in increased actin-myosin crosslinking and bundling that could lead to a stabilized actin cortex in adherent and suspended cells. There was no significant reduction in cell viability or increased PARP cleavage observed with the Rho Activator II dose used for this study, ruling out drug-related cytotoxicity as a result of ROCK over-activation (Supplementary Figure 2A&B).

### Modulating Rho/ROCK activity regulates the formation of microtentacles

**Figure 3:**
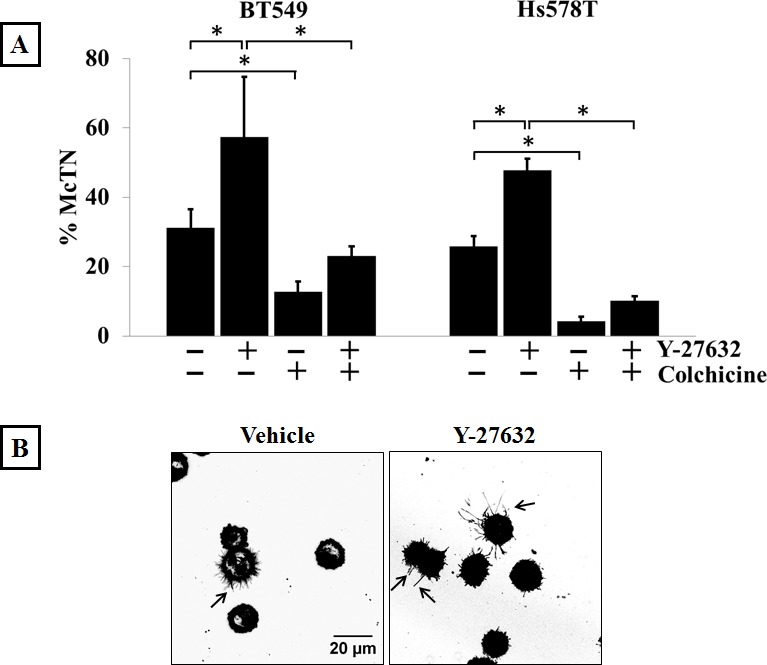

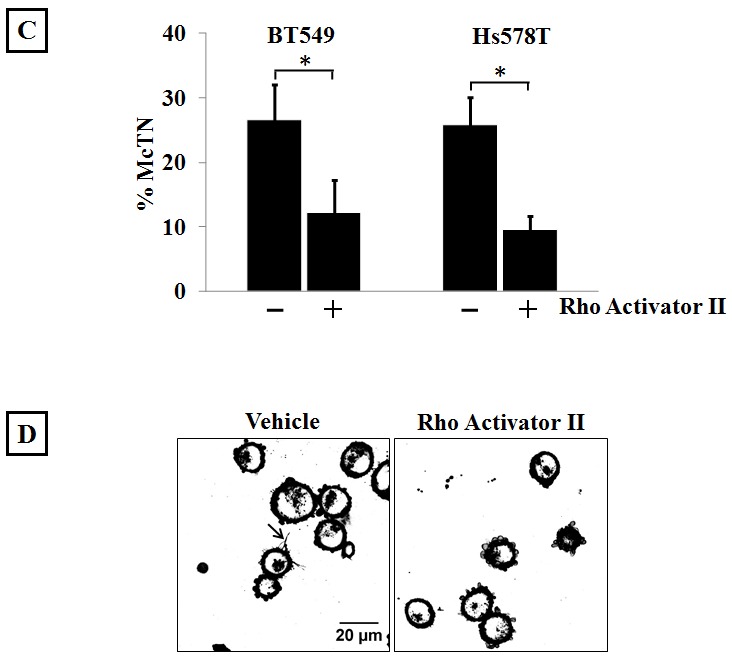
Modulation of Rho/ROCK activity regulates McTN formation A) Suspended BT549 and Hs578T cells stained with CellMask orange and scored blindly for the presence of McTNs show that 10μM Y-27632 treatment for 1 hour significantly increases the frequency of McTNs compared to vehicle-treated cells in both the cell lines tested. Treatment with 50μM colchicine, a microtubule-depolymerizing drug, significantly decreases McTN formation when used singly or in combination with Y-27632. Columns represent the mean +/− SD of three independent experiments in each of which 100 cells were scored in triplicate (900 cells total for every treatment group) (p<0.05, t-test, black asterisks). B) Representative live-cell confocal microscope maximum intensity projection images of CellMask orange stained suspended BT549 cells show more cells with McTNs in Y-27632-treated group compared to the vehicle control group (arrows) (60x magnification). C) 4μg/ml Rho Activator II treatment for 3 hours decreases McTN formation in BT549 and Hs578T cells. Columns represent the mean +/− SD of three independent experiments in each of which 100 cells were scored in triplicate (900 cells total for every treatment group) (p<0.05, t-test, black asterisks) D) Representative live-cell confocal microscope maximum intensity projection images of CellMask orange stained suspended BT549 cells show loss of McTN formation in Rho Activator II-treated cells compared to vehicle-treated cells (60x magnification).

We have previously published that modulation of the polymerization dynamics of the actin cortex can make cells more susceptible to the formation of McTNs [36]. Additionally, altering the activity of actin-destabilizing proteins such as cofilin can also effect McTN formation [37]. Given that Y-27632 treatment can decrease cellular contraction [31] we tested the effects of modulating ROCK activity on McTN formation. To determine if destabilization of the actin cortex as a result of ROCK pathway inhibition would increase McTN formation, BT549 and Hs578T cells were treated with 10μM Y-27632 for one hour, then detached and stained with a cell membrane binding dye for 10-15 min in an ultra-low attachment plate. After being blinded to the treatment groups, 100 cells from each group were scored for the presence of McTNs, with cells showing protrusions longer than the radius of the cell being considered positive. There was a significant increase in the percentage of cells that scored positive for McTNs in the Y-27632 treated group compared to those treated with vehicle control (Figure 3A&3B). As an additional control, the cells were also treated with the microtubule-depolymerizing drug colchicine. 50μM colchicine treatment for an hour dramatically reduced the frequency of McTNs compared to vehicle-treated cells but did not completely abolish McTN formation (Figure 3A). In order to ensure that the increased protrusions observed in the Y-27632 treated cells were microtubule-based; cells were treated with 50μM colchicine in addition to 10μM Y-27632. Colchicine significantly reduced the McTNs that were promoted by Y-27632, indicating that intact microtubule polymers are required for the formation of McTNs in the ROCK inhibitor treated cells (Figure 3A). Colchicine treatment did not completely block McTNs, so Y-27632 could still induce a relative increase by disrupting the actin cortical barrier, and allowing any residual microtubules to extend into McTNs.

Since a decrease in actin cortical integrity resulted in an increase in McTNs, and given the ability of Rho/ROCK to increase cellular contraction [31], we wanted to test whether Rho pathway over-activation could affect McTN formation. According to the cellular tensegrity model, compressional forces applied by the actin cortex result in buckling and breaking of microtubules when they encounter the cortical barrier [38]. BT549 and Hs578T cells were serum starved for 24 hours and then treated with 4μg/ml Rho Activator II and scored for McTN positivity. Both cell lines showed a significant decrease in the percentage of cells that scored positive for McTNs suggesting that increased cortical stability can inhibit the formation of McTNs (Figure 3C). Confocal live-cell images of suspended BT549 cells stained with a fluorescent cell membrane dye show McTNs on multiple cells in the Y-27632 treated group compared to the vehicle-treated group (Figure 3B). In contrast, Rho Activator II treatment decreases the number of cells presenting McTNs compared to the vehicle-control treated cells (Figure 3D).

### ROCK inhibition does not increase McTNs through microtubule stabilization

**Figure 4:**
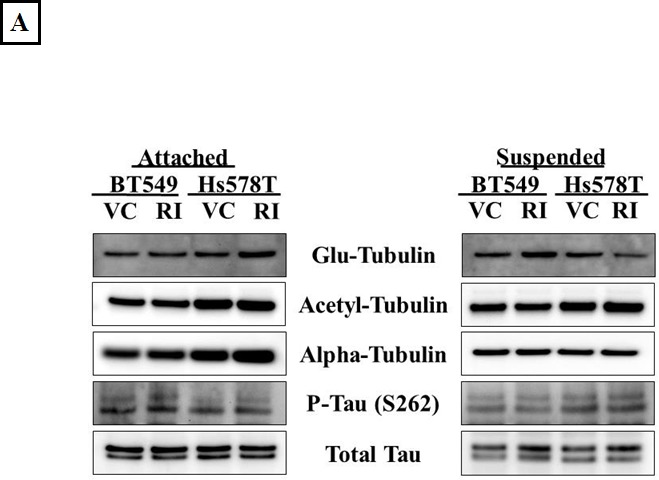

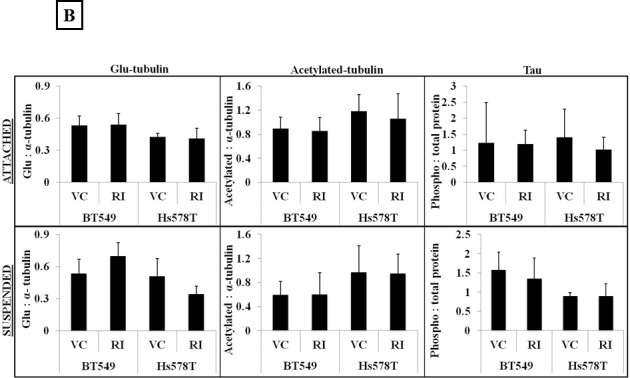

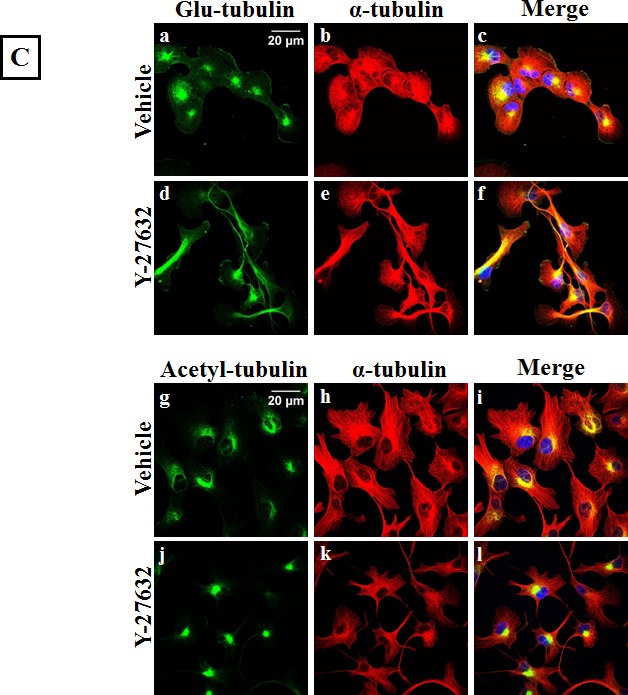
ROCK inhibition does not increase microtubule stabilization A) Representative western blot images (N=3) of attached and suspended BT549 and Hs578T cells and B) Densitometric analysis of 3 independent experiments show no significant change in ROCK-regulated markers of tubulin stability such as glu-tubulin (detyrosinated tubulin), acetylated tubulin or phosphorylated tau on treatment of the cells with 10μM Y-27632 [VC= Vehicle, RI=Y-27632] C) Immunofluorescence analysis of fixed BT549 cells stained for α-tubulin post-translational modifications; detyrosinated (glu) tubulin (top panel-a,d) and acetylated tubulin (bottom panel-g,j) did not show significant change in the distribution or localization of either protein on 10μM Y-27632 treatment; α-tubulin specific stain was used to indicate total tubulin levels in the BT549 cells (b,e and h,k) and nuclei were stained with Hoescht 33342 (60x magnification).

Apart from modulating the actin cytoskeleton, the Rho/ROCK pathway can also regulate microtubule stability through post-translational modifications such as acetylation, detyrosination and tau phosphorylation [13, 15, 16]. Given our previous data that microtubule stabilization can result in increased McTN frequency [26], ROCK-regulated markers of tubulin stability were analyzed to investigate the role of microtubule stabilization in McTN formation in the Y-27632 treated cells. ROCK has been reported to destabilize microtubules by decreasing tubulin acetylation [16] and by increasing tau phosphorylation, thereby preventing tau from binding and stabilizing microtubule filaments [15]. Although an increase in acetylated tubulin and a decrease in phosphorylated tau after Y-27632 treatment has been previously reported, we did not observe any significant change in acetylation of alpha-tubulin or tau phosphorylation upon ROCK inhibition in the breast cancer cells tested in adherent or suspended conditions (Figure 4A&B). Immunofluorescence analysis of Y-27632 treated BT549 cells showed a slight shift in the distribution of acetylated tubulin, but also overall changes in cell shape given the drug's effect on actin (Figure 4C), so it remains possible that the reorganization of acetylated tubulin merely reflects the change in cell morphology rather than any tubulin-specific reorganization. While ROCK works to destabilize microtubules, a different downstream effector of the Rho pathway, mDia increases microtubule stability by forming a complex that caps microtubule plus ends and promotes the formation of stable detyrosinated microtubules (also known as glu-microtubules) [39]. Since Y-27632 selectively targets the Rho effector ROCK but not mDia, we had predicted an increase in detyrosinated tubulin after Y-27632 treatment. However, densitometric analysis of 3 independent blots did not show a significant change in detyrosination of alpha-tubulin in adherent or suspended cells (Figure 4A&B). Additionally, immunofluorescence analysis did not show any significant changes in the localization or distribution of detyrosinated tubulin in the Y-27632 treated cells (Figure 4C). These results indicate that in the cell lines tested, inhibiting ROCK does not increase microtubule stability, suggesting that increased McTNs observed upon ROCK inhibition do not arise from increased microtubule stabilization.

### Inhibiting ROCK increases tumor cell reattachment

**Figure 5:**
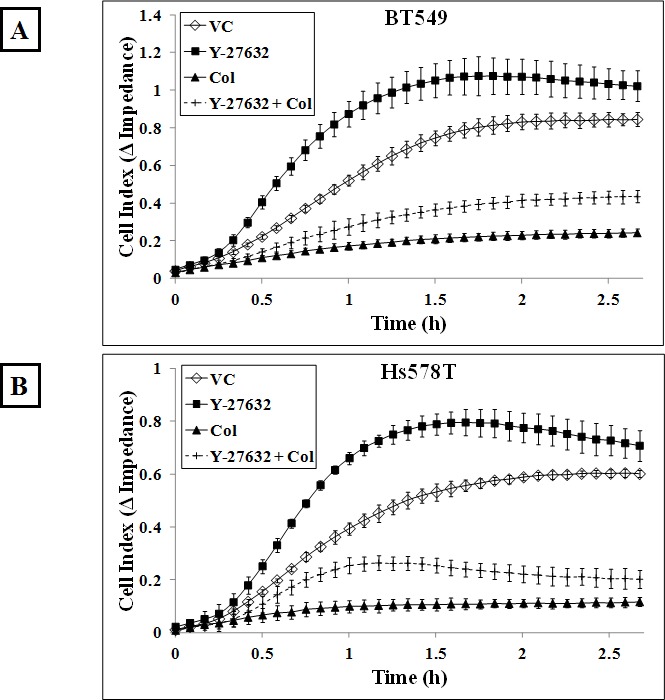

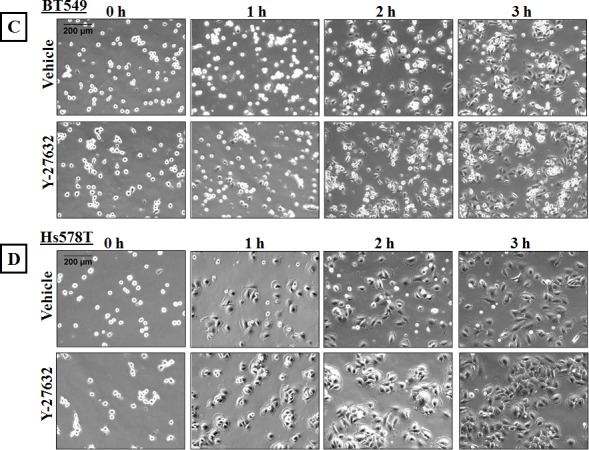
ROCK inhibition increases *in vitro* reattachment Real-time changes in electrical impedance caused by reattaching A) BT549 and B) Hs578T cells indicate an increase in reattachment efficiency of cells treated with 10μM Y-27632 for an hour compared to vehicle-treated cells. 50μM colchicine treatment for an hour decreases reattachment frequency following detachment when used singly or in combination with Y-27632 in both the BT549 and Hs578T cells. Graph represents mean +/− SD of triplicate values for each treatment group. Three independent experiments were performed. Representative phase contrast images of C) BT549 and D) Hs578T cells show cells reattaching to the plate more efficiently in the Y-27632-treated group compared to the vehicle-treated group (10x magnification) [VC= Vehicle, Col=Colchicine].

We have previously demonstrated that cells with higher McTN frequencies can reattach from suspension more efficiently than cells with lower McTN frequencies [40] and we wanted to determine whether Y-27632 treatment could similarly affect cell reattachment. The xCELLigence Real Time Cell Analyzer was used to assess the functional role of increased McTNs in the ROCK inhibitor treated cells. Using this device, reattachment is measured as impedance to the flow of a low-voltage electric current as cells reattach to the plate from suspension. BT549 cells that were treated with 10μM Y-27632 for one hour reattached more efficiently following detachment as compared to vehicle-treated cells (Figure 5A). When the cells were pre-treated with the microtubule-depolymerizing drug colchicine, the cells reattached significantly less than the vehicle-treated cells, indicating microtubules are essential for initial reattachment. In fact, addition of colchicine to the Y-27632 treated cells abolished the faster reattachment frequencies observed with Y-27632 treatment alone indicating that microtubule disruption by colchicine prevents increased reattachment even in cells with destabilized actin cortex. The same trend was observed in the Hs578T cell line (Figure 5B). To visualize the reattachment, phase-contrast images of cells were taken at corresponding time-points. More cells had attached to the plate in the Y-27632 treated group at earlier time-points compared to vehicle-control group (Figure 5C&D and Supplementary Figure 3A&B).

### Rho pathway over-activation decreases cell reattachment

**Figure 6:**
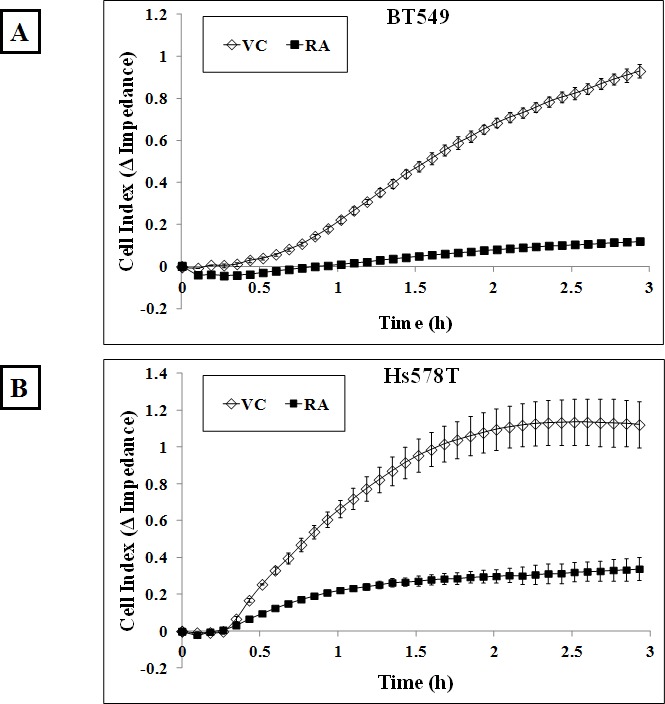

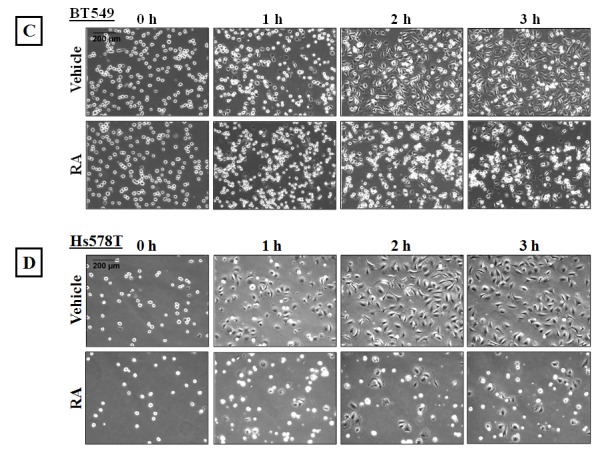
Rho over-activation decreases *in vitro* reattachment A) BT549 and B) Hs578T cells show a decrease in reattachment efficiency of 4μg/ml Rho Activator II-treated cells [RA] as indicated by the lower electrical impedance caused by reattaching cells compared to vehicle-treated cells [VC]. Graph represents the mean +/− SD of triplicate values for each treatment group. Three independent experiments were performed. Representative phase contrast images of C) BT549 and D) Hs578T cells reattaching to the plate show cells reattaching at earlier time-points in vehicle-treated controls compared to Rho Activator II-treated cells [RA] (10x magnification).

The actin cortical network applies a compressional force on the cell and this tension is resisted by microtubules, so that increased cortical tension prevents microtubules from pushing outwards [38]. We established that 4μg/ml Rho Activator II treatment promotes changes in the actin cytoskeleton and decreases McTN frequency. To assess the effect of reduced McTNs in Rho over-activated cells on reattachment efficiency, BT549 and Hs578T cells were serum starved for 24 hours and then treated with either vehicle or 4μg/ml Rho Activator II for 3 hours, detached and their reattachment was measured in real-time. The Rho Activator II treated cells showed a significant decrease in reattachment compared to vehicle-treated cells (Figure 6A&B). Phase-contrast images of the cells show that the control cells began the reattachment process by 1 hour and have almost completely attached by 3 hours while most of the Rho Activator II treated cells remain rounded even at 3 hours and very few reattach to the plate at the experimental endpoint (Figure 6C&D). These results confirm that modulation of the actin cortex through Rho activation can significantly decrease suspended cell reattachment.

## DISCUSSION

Cell migration and invasion are steps that are critical for metastasis and rely on Rho/ROCK-mediated cytoskeletal modifications and actomyosin contraction [41]. The ability of ROCK inhibitors to reduce the migratory and invasive properties of adherent tumor cells has led to suggestions that they could possibly be used to reduce metastasis in cancer patients and combined with the clinical success of ROCK inhibitors in other pathological conditions with deregulated actomyosin contraction such as cardiovascular diseases [42], hypertension [43] and atherosclerosis [44], ROCK inhibitors are gaining popularity as safe compounds that could be easily transitioned to cancer therapy.

However, in this study we show that ROCK inhibition increases the formation of microtentacles in detached breast tumor cells. As McTNs are only observed in cells that are detached and free-floating, they have not been previously reported in studies that have primarily focused on the effects of ROCK inhibitors in adherent cells. *In vivo* studies with colon carcinoma cells has demonstrated a microtubule-based mechanism for the reattachment of circulating cells to the capillary walls that is further enhanced by actin depolymerization [45]. This corresponds with previously published studies from our laboratory which have shown that altering the cytoskeleton with actin-destabilizing drugs or microtubule-stabilizing drugs results in an increase in the formation of McTNs in detached cells and enhances their reattachment [27, 30, 36].

In the present report, we show that in attached cells, destabilizing actin cortical integrity with the ROCK inhibitor, Y-27632, relaxes the cortex and makes the cells more elongated in appearance. The effects of Y-27632 on cell-shape deformation is enhanced in detached cells that do not experience any external cell-cell or cell-ECM contact resistance to cell shape deformation. In these cells, destabilizing actin filaments and reducing actomyosin contraction with Y-27632 abolishes the inward compressional force applied by the actin cortex. Without a barrier to buckle and break the outwardly-pushing microtubules, these cells show a significant increase in the formation of McTNs. Further proof of the concept that microtubule outgrowth is antagonized by actin cortex is the reduction in the frequency of McTNs observed in cells treated with the Rho activator, Rho Activator II. Although detachment usually induces formation of McTNs in cells, increasing actomyosin cross-linking and contractility counteracts this effect in the suspended Rho activated cells.

The Rho/ROCK signaling pathway also regulates the microtubule cytoskeleton and Y-27632 treatment has been reported to increase microtubule stability [16, 46]. However we did not observe consistent changes in markers of tubulin stability such as tau phosphorylation, or changes in alpha-tubulin post-translational modifications such as detyrosination or acetylation. While it is possible that the duration of treatment or concentration used was not sufficient to cause the organizational changes in the breast cancer cells used, our results suggests that the increase in McTNs observed with Y-27632 treatment is largely due to its effect of destabilizing the actin cortex, rather than a result of microtubule stabilization

Disseminated tumor cells in the bone marrow and circulating tumor cells in the blood are rapidly emerging as markers that can predict patient clinical outcome [23] and there are currently a number of interventional clinical trials underway to investigate the benefits of using CTCs as a prognostic marker to determine treatment options [47]. Most current and developing chemotherapeutics focus on reduction in cell proliferation, migration and invasion to determine drug efficacy and thus fail to address the effects of these drugs on free-floating cells that are in circulation. ROCK inhibitors have been reported as efficacious anti-cancer agents based on studies done in attached cell conditions, but our study shows that they could promote reattachment of circulating tumor cells by increasing McTN formation in detached cells. A number of studies in the last decade have shown a positive correlation between high CTC levels and metastatic disease [22] highlighting the importance of targeting CTCs. Nevertheless, many current clinical regimens using traditional chemotherapeutics may fail to eradicate CTCs, which are often quiescent and thus are resistant to chemotherapeutics that target cell division [48]. Even newer targeted tumor-specific small-molecule inhibitors or antibody therapies may be ineffective on CTCs if the CTCs are molecularly different from the primary tumor cells as a result of newly acquired mutations or selective dissemination of clonally heterogeneous tumor cells [23]. For example, microtubule-targeting cytotoxic chemotherapies such as taxanes, which are extremely effective in their anti-proliferative effects on the primary tumor, can significantly increase CTCs in the patients' blood when given prior to surgery [49]. Accumulating evidence on the role of CTCs in promoting metastasis [22-24] highlights the necessity of screening developing chemotherapeutics for their effects on free-floating suspended cells in addition to adherent cell conditions to avoid inadvertently increasing CTCs and metastatic risk.

Recently a number of studies have found EMT and stem cell markers in CTCs and these cells have been found to have higher metastatic efficiency [50]. Interestingly, a number of recent studies have shown that use of Y-27632 in routine culture promotes survival and proliferation of dissociated human embryonic stem cells by preventing dissociation-induced apoptosis [51-53]. Moreover, organizational changes in the actin cytoskeleton and inhibition of actomyosin contractility regulate stem cell fate of human keratinocyte stem cells by preventing clonal conversion [54]. Intriguingly, inhibition of actomyosin contractility promotes induction of stem-like phenotype in primary cancer cell lines [55]. Liu *et al* show that this phenomenon is not due to selective survival of rare stem-like cells present in the population but rather a result of conditional reprogramming of cells and use a combination of Y-27632 and feeder cells to indefinitely proliferate cultures generated from as few as 4 tumor cells [56]. The applications of inhibition of contractility in stem cell generation and proliferation is important for diagnostic and regenerative medicine. But at the same time, it also calls to attention the possible implications of using ROCK inhibitors as cancer therapeutics if they promote survival and possibly conversion of detached tumor cells towards a stem-like phenotype. We have recently reported that breast cancer cell lines with higher stem-like characteristics have increased McTNs that promote their reattachment from suspension [57]. These findings indicate that chemotherapeutics that inadvertently promote such stem-like characteristics might be detrimental to overall progression-free survival.

Disseminated tumor cells in circulation are predicted to be precursors of distant metastasis and therefore, new drugs that are being developed as potential cancer therapeutics need to be examined for their effect on CTCs. The biggest challenge CTC research faces is that CTCs are present at very low numbers in the blood; on the order of 1 CTC per 10^8^ blood cells, making it very difficult to both detect CTCs and obtain large enough numbers to test for drug efficacy. While the low number of recoverable viable CTCs prevents many functional studies, one alternative is to mimic the microenvironment of CTCs, which should at least include free-floating cell conditions when testing drug effects. The vast majority of studies evaluating the efficacy of ROCK inhibitors as cancer therapeutic focus on adherent cell behavior and have failed to address their effect on disseminated tumor cells that are in circulation. In this study we show that ROCK inhibition in detached cells can increase McTN formation and *in vitro* reattachment efficiency. Since ROCK inhibitors might prove useful in reducing primary tumor dissemination because of their anti-migratory and anti-invasive effects, in order to maximize their anti-cancer efficacy, it might be prudent to use ROCK inhibitors in conjunction with microtubule targeting drugs to reduce McTNs [40] and thereby prevent inadvertently increasing metastatic risk. Our results emphasize the importance of considering the current attachment state of circulating tumor cells in patients, so that targeting ROCK to reduce the migration and invasion of adherent tumor cells does not inadvertently increase the metastatic potential of non-adherent circulating tumor cells.

## MATERIALS AND METHODS

### Cell culture and reagents

BT549 and Hs578T cells were cultured in DMEM (Corning Cellgro) with 10% fetal bovine serum and 1% Penicillin/Streptomycin and maintained at 37°C in 5% CO_2_. Y-27632 and Rho Activator II (CN03) were obtained from Cytoskeleton, Inc. The Rho Activator II drug concentration used for this study was determined based on the dose at which maximum stress fiber formation was observed, as recommended by the manufacturer. Primary antibodies used for immunoblotting and/or immunofluorescence were obtained from Cell Signaling Technology for P-MYPT1 (T853), Total-MYPT1, P-MLC (S19), Total-MLC, P-cofilin (S3), Total-cofilin and Acetyl (L-40) tubulin; Abcam for P-tau (S262) and detyrosinated-tubulin; and alpha-tubulin was obtained from Sigma.

### Immunofluorescence

For fixed cell imaging, cells were plated on glass coverslips placed in a 24-well dish. After appropriate drug treatments, cells were fixed with 3.7% paraformaldehyde (10min), permeabilized with 0.25% Triton X-100 in PBS solution (10min) and blocked with 5% BSA/0.5% NP-40 in PBS (1hour). Cells were incubated overnight at 4°C with primary antibodies (1:500 dilutions) in 2.5%BSA/0.5%NP-40 in PBS solution. Cells were subsequently washed with PBS and incubated with corresponding anti-Mouse or anti-Rabbit Alexa-Flour 488 or 568 conjugated secondary antibodies (1:1000 dilutions) and Hoechst 33342 (1:5000; Invitrogen) in PBS for an hour at room temperature. To visualize filamentous actin, cells were stained with phalloidin Alexa-Fluor 488 (1:500; Molecular Probes) in PBS for an hour at room temperature. Cells were washed with PBS and the coverslips were mounted on microscope slides. Images were taken using the Olympus FV1000 laser scanning confocal microscope.

### Immunoblotting

Cells were treated with appropriate drugs (10μM Y-27632 for 1 hour; 4μg/ml Rho Activator II for 3 hours) and whole-cell lysates were harvested using 4x laemmli buffer. The lysates were passed through a 25 gauge syringe 10 times and then boiled at 95°C for 7-8 minutes. Protein concentrations were quantified using DC protein assay (Biorad) and lysates were diluted with water to a 2x buffer concentration prior to loading. DTT (final concentration 0.1mM) and 1ul of 0.4% bromophenol blue were added to the lysates and 20μg of the lysates were run on 4% to 12% NuPage MES Bis-Tris gradient gel (Invitrogen) and transferred onto polyvinylidene difluoride membranes. The blots were blocked with 5% milk or 5% BSA (for the phospho-antibodies) for an hour and probed with primary antibodies (1:1000) overnight at 4°C. The blots were subsequently incubated with horseradish peroxide (HRP) conjugated secondary antibodies (1:1000, Jackson ImmunoResearch) for an hour. Densitometric analysis of 3 independent experiments was done using ImageJ software and statistical significance was computed using the student's t-test.

### McTN scoring and imaging

BT549 and Hs578T cells were treated for an hour with either vehicle; 10μM Y-27632; 50μM colchicine or a combination of 10μM Y-27632 and 50μM colchicine. Prior to treatment with the Rho activator, cells were serum-starved for 24 hours and then treated with either vehicle or 4μg/ml Rho Activator II for 3 hours. After appropriate drug treatments, cells were trypsinized and the detached cells were pelleted by centrifugation (1000 rpm, 5min). Cell pellet was resuspended in phenol-red free DMEM containing 1:2500 CellMask orange (Invitrogen) and incubated for 10-15 min at 37°C and 5% CO_2_ in ultra-low attachment plates. After being blinded to the treatment groups, 100 cells were scored from each group for the presence of McTNs, with cells showing protrusions longer than the cell radius being counted as positive. Each treatment group was scored in triplicate and 3 such independent trials were conducted to generate mean and SD of McTN counts (total of 900 cells for each treatment group). Statistical significance was verified using the student's t-test. To generate the McTN images used in the manuscript, the CellMask stained live cells were imaged using the Olympus FV1000 laser scanning confocal microscope. Stacked images were converted to maximum intensity projection using ImageJ.

### Attachment assay

Cells were treated for an hour with either vehicle; 10μM Y-27632; 50μM colchicine or a combination of 10μM Y-27632 and 50μM colchicine. Prior to treatment with the Rho activator, the cells were serum-starved for 24 hours and then treated with either vehicle or 4μg/ml Rho Activator II for 3 hours. Following appropriate drug treatments, cells were trypsinized and counted. 2×10^4^ cells were plated in triplicate into the RTCA xCELLigence microwell E-plates (ACEA Biosciences). The microwell plates used for this assay have electrodes that can measure the change in the flow of low-voltage electric current passing through the plate. As cells attach to the plate there is resistance to the flow of current and this change in impedance is a direct measure of number of cells that have adhered. After adding the cells, the plate was placed in the RTCA DP Analyzer located in an incubator maintained at 37°C and 5% CO_2_. Cell reattachment to the plate was measured as perturbation to the flow of current passing through the E-plate and a reading was taken every 5 minutes for 3 hours. The change in impedance to the flow of current over time was used to determine the reattachment efficiency of the cells. Each treatment group was run in triplicate and the graph represents the mean +/− SD. Three such independent trials were conducted.

### Cell viability

Cell viability was measured using the CellTiter 96® AQ_ueous_ One Solution Cell Proliferation Assay (Promega) as per manufacturer instructions. Briefly, 2×10^4^ cells were plated in triplicate in a 96well plate and allowed to adhere for 24 hours. For Rho activator treatment, cells were incubated in serum-free media for 24 hours prior to drug treatment to mimic experimental conditions followed for assays mentioned previously. Cells were treated with appropriate drugs and then incubated with the CellTiter reagent for 2 hours at 37°C and 5% CO_2_. Absorbance was measured at 490nm and values obtained were averaged and normalized over vehicle-control. 3 such independent trials were conducted to obtain mean +/− SD.

## SUPPLEMENTARY MATERIALS FIGURES


