# Supplementary material for: ROCK inhibition promotes microtentacles that enhance reattachment of breast cancer cells
Source: Oncotarget. 2015 Jan 31;6(8):6251–66. doi: 10.18632/oncotarget.3360 (PMC4467435; doi:10.18632/oncotarget.3360)
Supplement: Supplementary file 1 [file oncotarget-06-6251-s001.pdf]

ROCK inhibition promotes microtentacles that enhance reattachment of breast cancer cells

Supplementary Material

A

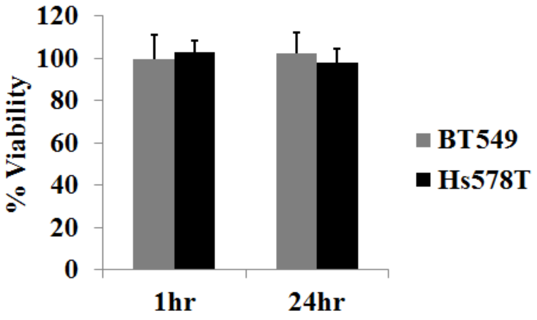

B

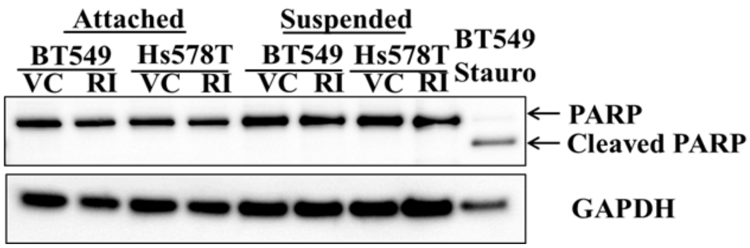

Supplementary figure 1: Y-27632 treatment does not affect cell viability
A) Cell viability measured using a metabolic colorimetric assay; CellTiter (Promega) shows no decrease in viability on treatment of BT549 and Hs578T cells with 10µM Y-27632 for 1 hour or 24 hours. Columns represent averaged values of 3 independent experiments done in triplicate, normalized over vehicle-control B) Representative western blot image of attached and suspended BT549 and Hs578T cells treated with 10µM Y-27632 [RI] for 1 hour shows absence of PARP cleavage (N=3). BT549 cells treated with 0.5µM staurosporine for 1 hour was used as a positive control for PARP cleavage.

A

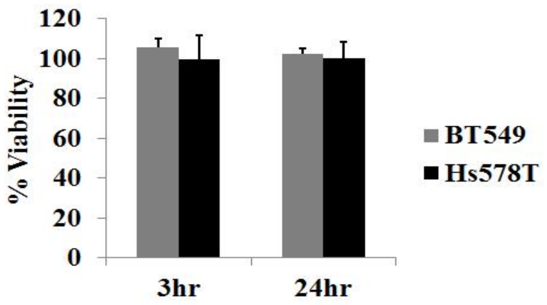

B

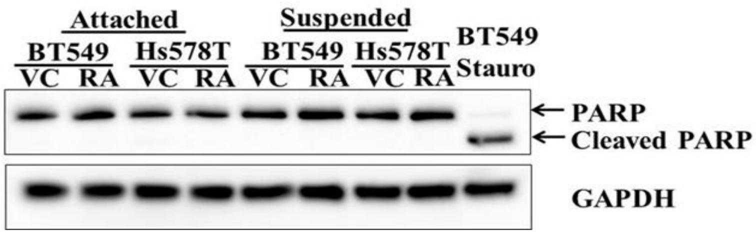

Supplementary figure 2: Rho Activator II treatment does not affect cell viability

A) Cell viability measured using a metabolic colorimetric assay; CellTiter (Promega) shows no decrease in viability of BT549 or Hs578T cells on treatment with 4µg/ml Rho Activator II for 3 hours or 24 hours. Columns represent averaged values of 3 independent experiments done in triplicate, normalized over vehicle-control. B) Representative western blot image of attached and suspended BT549 and Hs578T cells treated with 4µg/ml Rho Activator II [RA] for 3 hours shows absence of PARP cleavage (N=3). BT549 cells treated with 0.5µM staurosporine for 1 hour was used as a positive control for PARP cleavage.

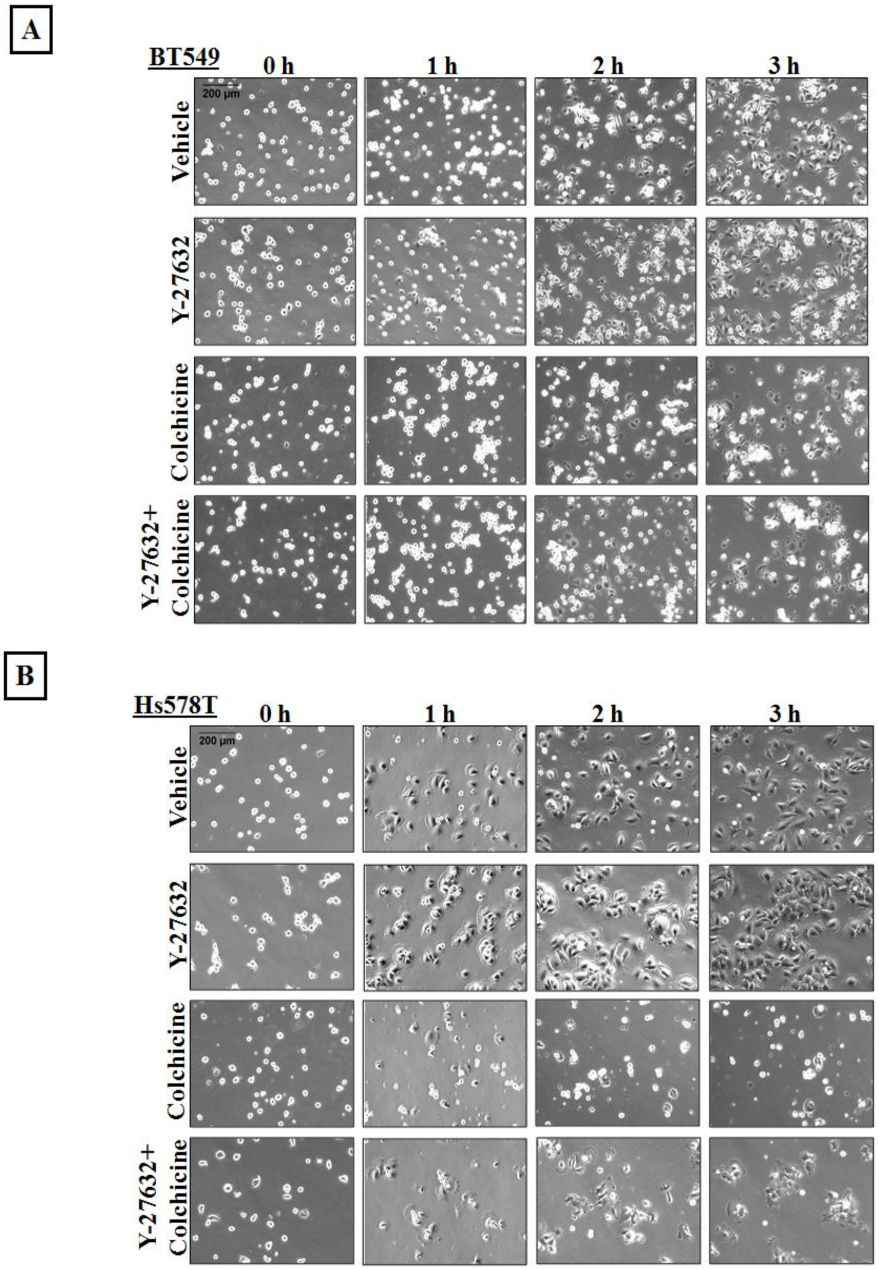

Supplementary figure 3: Colchicine treatment reduces reattachment of cells from suspension

Phase contrast images of A) BT549 and B) Hs578T cells treated for an hour with vehicle control or 10µM Y-27632 or 50µM colchicine or a combination of 10µM Y-27632 and 50µM colchicine show differences in reattachment efficiencies of cells from each treatment group following detachment. Cells treated with 10µM Y-27632 reattach more efficiently compared to vehicle-treated group. 50µM colchicine treatment significantly reduces the reattachment frequency of cells when used singly or in combination with Y-27632 (10x magnification).
